# A Comparison of the Mediterranean Diet and Current Food Patterns in Italy: A Life Cycle Thinking Approach for a Sustainable Consumption

**DOI:** 10.3390/ijerph191912274

**Published:** 2022-09-27

**Authors:** Giuliana Vinci, Lucia Maddaloni, Sabrina Antonia Prencipe, Marco Ruggeri, Maria Vittoria Di Loreto

**Affiliations:** Department of Management, Sapienza University of Rome, Via del Castro Laurenziano 9, 00161 Rome, Italy

**Keywords:** Mediterranean diet, food patterns, Italian consumption: sustainability, LCA, LCT, carbon footprint, CED

## Abstract

The transition toward more sustainable food systems, which already represents a central element of the European Farm to Fork and Green Deal strategies, could be an effective measure to contribute to global decarbonization and greenhouse gas (GHGs) reduction goals; concurrently, it could improve the health status and nutrition of the global population. In this context, the Mediterranean diet (MD) could play a considerable role, as it is generally recognized as a more balanced, healthy, and sustainable eating pattern than Western consumption patterns, which are characterized by excess food and high energy content, thus causing undesirable effects on both human health and the environment. Although traditionally linked to MD, Italy sees relatively moderate adherence by its citizens, as they consume about +75% of the daily caloric intake recommended by MD. Therefore, this study aims to quantitatively assess the potential environmental, economic, and health impacts of this lower adherence to MD by Italians. Current Italian Food Patterns (CIFP) in 2019 were analyzed and compared to the MD recommended amounts through a Life Cycle Thinking (LCT) approach (LCA) and carbon footprint (CF) analysis. The results show that CIFP, compared to MD, has +133% greater impacts on the environmental macro-area, +100% greater impacts on the human health macro-area, and +59% greater impacts on the economic macro-area (with annual fossil and mineral resource savings of $53.35 per person, $3.2 billion per year). The analysis also shows that CIFP has a CF of 6.54 × 10^1^ kg CO_2_ eq, +142% over MD (2.7 × 10^1^ kg CO_2_ eq), resulting in a lower environmental impact of the Mediterranean diet.

## 1. Introduction

Food production accounts for approximately 21–37% of anthropogenic greenhouse gas emissions (GHGs) globally [1], mainly due to changes in Western eating habits, driven by a progressive shift away from traditional diets characterized by plant-based foods rich in fiber, complex carbohydrates, and micronutrients [2]. This resulted in an increased preference for diets based on higher consumption of animal products and rich in refined fats and sugars [3]. Frequent consumption of nutritionally unbalanced meals has led to an increase in obesity (currently affecting about 500 million people) and overweight (1.5 billion people) [4,5], which in turn could induce a higher incidence of cardiovascular disease [6], responsible globally for about 41 million deaths each year [7]. Therefore, the shift toward healthier and more sustainable dietary patterns could both optimize food supply chains and improve the health and nutrition of the global population [8]. Among the proposed models for healthy diets and sustainable consumption, the Mediterranean diet (MD) prevails [9], thus representing a healthy and balanced UNESCO heritage food model based on frequent consumption of fruits and vegetables, grains and legumes, nuts, and low consumption of red meat, with extra virgin olive oil as the main source of fat [10]. The sustainability assessment of different nutritional models is gaining international interest in scientific literature. Castañé and Antón (2017) [11] used Nutrient Food Rich (NFR) 9.3 and life cycle assessment (LCA) to investigate the nutritional quality and environmental impacts of MD versus vegan, with the former having a slightly lower nutritional score than the latter, but twice the global warming potential and three times the biodiversity impact. Walker et al. (2018) [12] looked at the typical diet of 1457 European adults and measured the impacts of food production for each individual, showing that food choices associated with dairy, meat, and vegetable consumption cause greater impacts on human health than consumption of vegetables and seeds due to their production processes. Instead, Esteve-Llorens et al. (2020) [13] evaluated the carbon footprint (CF) of the Portuguese dietary pattern (4.20 kg CO_2_ eq/inhabitant/day on average) in comparison with an alternative diet, which leads to an increase in the nutritional quality of about +67% and a CF of −25%. Finally, González-García et al. (2018) [14] compared the CF and water footprint (WF) and Life Cycle Costing (LCC) of three diets recommended in Spain, showing that the South Atlantic Diet generates a higher CF, WF, and LCC than the MD (+30%, +23%, and +21%, respectively) and the Spanish Dietary Guidelines (+15%, +9%, and +21%, respectively). Although Italy is traditionally linked to the Mediterranean diet [15], its current dietary pattern deviates significantly from the “Reference Intake Levels of Nutrients and Energy for the Italian Population (LARNs)” [16,17]. In fact, according to LARNs [17], the Average Dietary Energy Requirement for a normal-weight individual is estimated to be about 2000 Kcal/person/day, while in Italy, the population consumes an average of 3503 Kcal/person/day [4], +1503 kcal/day (+75% of calories compared to MD recommended levels) due to higher consumption of meat and meat products (+62%), animal fats (+32%), and sugars and sweeteners (+24%) [4,9]. Furthermore, it is worth noting that this requirement may vary based on a person’s age and weight [3,15]. A transition to more sustainable food systems could be an effective measure to contribute to the global decarbonization and GHGs reduction goals, which already represent central elements of the European Farm to Fork [18] and Green Deal [19] strategies and Sustainable Development Goals (SDGs) [20]. In the meantime, it could improve the health status and nutrition of the global population. In this framework, a sustainability assessment of diets could be important to consider both ecosystem impacts and direct and indirect health implications. Therefore, this study aimed to analyze the environmental, economic, and human health impacts caused by the production of foods routinely consumed by the Italian population in 2019 (pre-pandemic) compared to the MD-recommended amounts. In particular, two cases were considered and compared: the Current Italian Food Patterns (CIFP) for the year 2019, based on available data [21,22,23], and the quantities recommended by the MD guidelines. The final objective was to conduct a sustainability study related to non-adherence to MD by the Italian population. The impact assessment was carried out through an LCT approach, especially through LCA and CF, using SimaPro 9.2.2. software.

## 2. Materials and Methods

The LCA methodology was used to assess the long-term economic, environmental, and human health impacts of the 2019 Current Italian Food Patterns (CIFP) compared to the MD guidelines [17]. CIFP refers to the amount of food purchased by Italian consumers. Part of this amount is consumed during main meals, contributing to daily energy requirements, and the other part is destined to become food waste (FW) [24].

**Figure 1:**
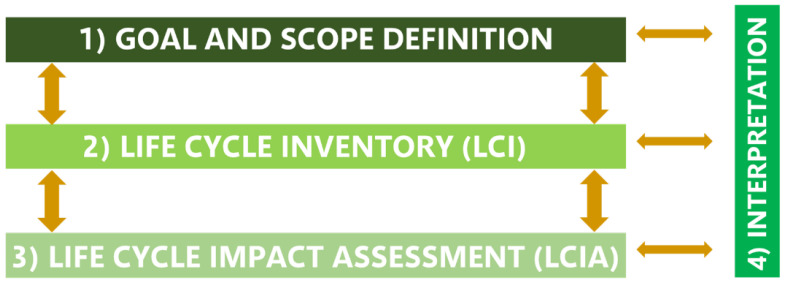
The four phases of the life cycle assessment.

LCA allows the evaluation of a product or service’s potential environmental, social, and economic impacts. It is a standardized method based on ISO 14040:2006 [25] and ISO 14044:2006 [26]. As shown in Figure 1, it consists of four stages: (i) Goal and scope definition; (ii) Life Cycle Inventory (LCI); (iii) Life Cycle Impact Assessment (LCIA); (iv) Interpretation.

The evaluation and comparison were conducted using the SimaPro 9.2.2 software. The data used for this study were processed based on those collected from Coldiretti [21], ISMEA Markets [22], and ISTAT [23]. Data processing was carried out considering the amount of the different food groups recommended by the LARN [17] expressed in g/per capita/week, including those foods whose intake frequency is equal to 1–2 times a week. Based on per capita consumption, expressed in kg/per capita/year, the weekly quantities (g/per capita/week) of the following foods were calculated: (i) meat (red and white meat, processed meat), fish (fresh and/or fish products) and eggs; (ii) milk and derivatives (milk, yogurt, fresh and aged cheeses); (iii) cereals/derivatives (bread and bread substitutes, pasta, products from oven, cakes) and tubers; (iv) legumes (fresh and/or dried); (v) seasoning fats (extra virgin olive oil); (vi) fruit (fresh and dried) and vegetables (cooked and raw); (vii) random foods (sweets, snacks, sugary drinks, sugar, and alcoholic beverages).

### 2.1. Goal and Scope Definition

The study aimed to assess the environmental, economic, and human health impacts of the industrial processes related to 2019 Current Italian Food Patterns (pre-pandemic consumption) compared to the Mediterranean diet model. The system boundaries of this study consist of a cradle-to-gate approach [27]. Therefore, the stages of production (cultivation, fishing), industrial processing, manufacturing, transport, packaging, retailing, and consumption were considered. Food waste disposal was not considered. The energy content of the diet was chosen as the functional unit (FU) in other studies, such as Castañé e Antón (2017) [11], Veeramani et al. (2017) [28], and van de Kamp et al. (2018) [29]. In particular, FU has been chosen as 14,000 kcal, the guideline-recommended weekly caloric intake for normal-weight adults, whose daily requirement amounts to about 2000 kcal/day [17].

### 2.2. Life Cycle Inventory (LCI)

**Figure 2:**
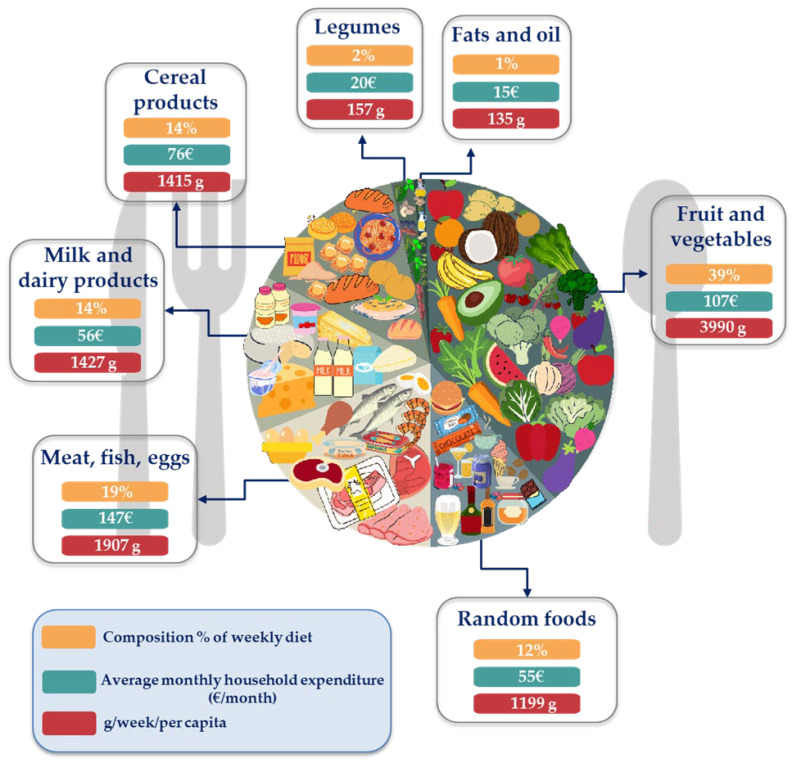
Weekly distribution of foods consumed in Italy (2019) by food groups (ISTAT, 2021).

The MD input data were processed based on the LARN (2018) [17], respecting the portions and the weekly consumption frequency for each product belonging to the different food groups. As for the national consumption, the quantity in g/per capita/week was obtained from the consumption data of kg/per capita/year [23], giving 10,230 g of food per week/per capita, divided as in Figure 2. The values obtained were then normalized to the functional unit of 14,000 kcal and used as input to compare the MD and Italian consumption (Regarding random foods, even if an occasional consumption is recommended, as 1–2 times a month, they have been considered in the weekly calculation of calories to represent the average better). Table 1 shows the input and output data for the Mediterranean nutritional model and the 2019 CIFP. Generic input processes and flow were obtained from Agribalyse v3.0.1 [30], Ecoinvent v3.8 [31], and World Food LCA Database (WFLDB) v3.5 [32] datasets.

### 2.3. Life Cycle Impact Assessment (LCIA)

The LCIA aims to identify and assess food products’ contribution to the impact categories considered. The methods used are “ReCiPe 2016 Endpoint” and “Cumulative Energy Demand (CED)”. Both methods consider the consequences that food production processes have on the environment and energy production due to the damage to pathways they cause [33].

In the case of the “ReCiPe 2016 Endpoint”, the impact categories analyzed were divided into three macro-areas: Environmental (Global warming, terrestrial ecosystems; Global warming, freshwater ecosystems; Ozone formation, terrestrial ecosystems; Terrestrial acidification; Freshwater eutrophication; Marine eutrophication; Terrestrial ecotoxicity; Freshwater ecotoxicity; Marine ecotoxicity; Land use; Water consumption, Terrestrial ecosystems; Water consumption, aquatic ecosystems); Human Health (Global warming; Stratospheric ozone depletion; Ionizing radiation; Ozone formation; Fine particulate matter formation; Human carcinogenic toxicity; Human non-carcinogenic toxicity; Water consumption); and Economic (Mineral resource scarcity; Fossil resource scarcity).

The impacts of the Environmental macro-area are expressed in species/year, which is a way to measure the extinction rate and the impact on biodiversity, estimating one extinction per million species/year. The impacts of the Human Health macro-area are expressed in “Disability-Adjusted Lost Years (DALYs)”, which is a measure chosen by WHO to estimate the “Global Burden of Diseases”, the overall severity (or overall impact) of disease [34]. It is expressed as the number of years lost due to illness, disability, or premature death. It extends the concept of potential life years lost due to premature death to include years of “healthy” life lost due to poor health or disability. In summary, it calculates how many years of healthy life have been lost due to a particular pathology caused by a specific problem, in this case, an impact category. The data of the economic macro-area are expressed in USD. Instead, the CED was applied to quantify the energy removed from nature by the two scenarios, a tool widely used to estimate the energy required by a good or service during its lifecycle. This does not replace an assessment with comprehensive impact assessment methods, such as ReCiPe 2016 or ILCD 2011, but it makes sense as an integrated approach to confirm the analysis data conducted with other methods.

### 2.4. Carbon Footprint (CF)

Based on the LCI and LCIA, it was possible to calculate the carbon footprint of the scenarios examined. Carbon footprint is a measure that expresses the greenhouse gas (GHGs) emissions generated by a product, service, organization, etc. Generally, it is expressed in kilograms of CO_2_ equivalent, and by the Kyoto protocol, Carbon Dioxide (CO_2_), Methane (CH_4_), Nitrous Oxide (N_2_O), Hydrocarbons, Hydrofluorocarbons (HFCs), Sulfur Hexafluoride (SF_6_), and Perfluorocarbons (PFCs) are taken as reference (IPCC, 2006). Each gas has a different greenhouse effect, so the CO_2_ equivalent was calculated according to Forster et al. (2007) [35] as follows:

Carbon footprint = ∑G.G.i × ki

where G.G.i is the amount of greenhouse gas produced and ki is the CO_2_ equivalent coefficient for that gas.

## 3. Results

LCA results are shown in Table 2.

**Figure 3:**
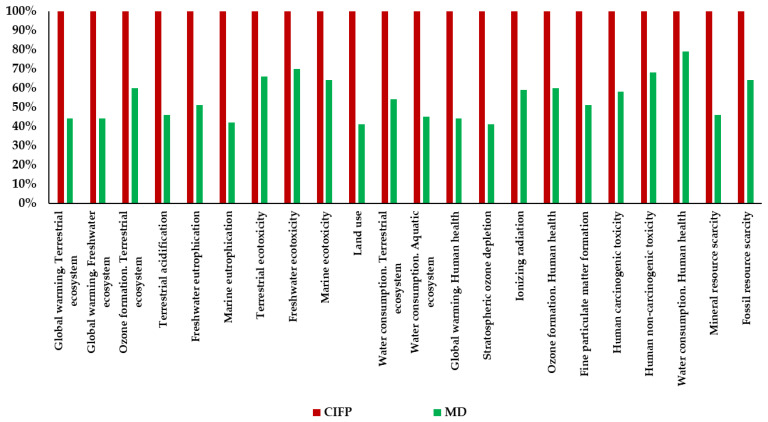
Different impacts of Italian consumption and the Mediterranean diet (MD: Mediterranean diet; CIFP: Current Italian food patterns).

The results were also characterized and expressed as relative impact (Figure 3), where the scenario with the highest value in the impact category is set as the reference value (100), and the others were calculated accordingly. This visualization allows for a better representation of LCA results, especially when using calculation methods such as the ReCiPe 2016 Endpoint, which considers impact categories with different units of measurement and then analyzes the categories on the same scale to highlight specific trends. Notably, both Table 2 and Figure 3 show how MD impacts are lower for all impact categories than those obtained for current consumption in Italy. For the macro area “Environment”, with 1.05 × 10^−6^ DALYs, CIFP generates an overall impact of +133% higher than that of the Mediterranean diet (4.52 × 10^−7^ DALYs) with the categories in which current consumption has a greater impact than the Mediterranean diet being “Land Use (LU)” (+145%), “Marine Eutrophication (ME)” (+135%), “Global warming, Terrestrial Ecosystem (GWTE)” (+127%), and “Global warming, Freshwater Ecosystem (GWFE)” (+127%), “Water consumption, Aquatic ecosystem (WCAE)” (+123%). Concerning the “Human Health” macro area, Italian dietary habits in 2019 had a more significant impact than those of the Mediterranean model by +100% (7.70 × 10^−5^ DALYs vs. 1.54 × 10^−4^ DALYs) with higher impacts in 8 out of 8 categories compared to the Mediterranean diet, with values ranging from +27% (Water consumption, human health), where CIFP presents values of 2.31 × 10^−6^ DALYs vs. 1.82 × 10^−6^ of MD, to +143% of “Stratospheric Ozone Depletion (SOD)”, where CIFPS affects 1.98 × 10^−7^ DALYs vs. 8.16 × 10^−8^ of MD.

Regarding the economic macro area, on the other hand, CIFP ($2.78) has a greater impact than MD ($1.75) by +59%. In this regard, it might be possible to calculate the kcal-related economic savings for each dietary pattern. For MD, the economic impact is $0.03 per MRS and $1.72 per FRS, for a total of $1.75 per capita per week. In CIFP, the economic impact is $2.78 in total ($2.71 for FRS and $0.07 for MRS), as shown in Table 3.

It was found that adopting MD over the current eating habits of Italians could result in monetary savings of $1.03 per capita per week. Considering 52 weeks, each Italian could induce an annual fossil and mineral resource savings of $53.35, generating a total savings of $3.2 billion per year compared to the current Italian population. Subsequently, the cumulative energy demand (CED) and carbon footprint were also calculated for a greater understanding of the results to confirm the LCA results.

As for CED (MJ equivalent), it was divided into two main categories (renewable and nonrenewable) and eight subcategories (fossil, nuclear, biomass, wind, solar, geothermal, and water), as shown in Table 4, from which it can be seen that current Italian dietary habits result in a higher share of fossil resource use (3, 24 × 10^2^ MJ eq for the current food model vs. 1.90 × 10^2^ MJ eq for the Mediterranean diet) due to emissions caused by diesel and fuels for transporting food commodities and the production and increased use of primary and secondary plastic packaging (polypropylene and polyethylene).

**Figure 4:**
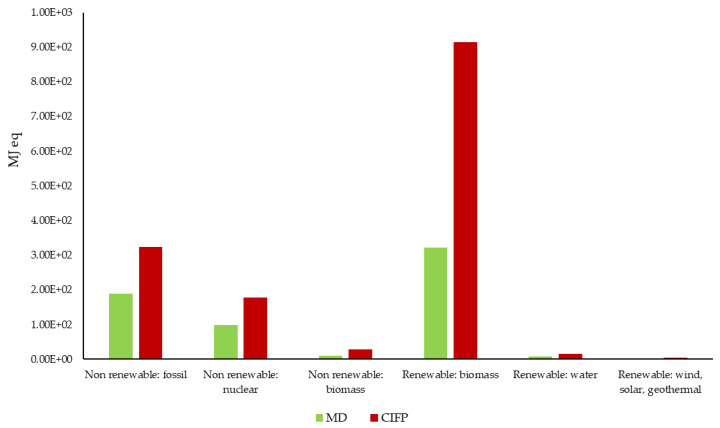
Cumulative energy demand of Mediterranean diet and Italian dietary habits (MD: Mediterranean diet; CIFP: Current Italian food patterns).

Italian food habits also result in higher use of energy sources from nuclear power plants (1.78 × 10^2^ MJ eq vs. 9.79 × 10^1^ MJ eq), which can be attributed to higher use of electricity. The characterized results (Figure 4) show that, in general, the Mediterranean diet is more sustainable than current dietary patterns, as it is less energy-intensive in categories related to the use of energy raw materials. This confirms the results obtained from the ReCiPe Endpoint.

**Figure 5:**
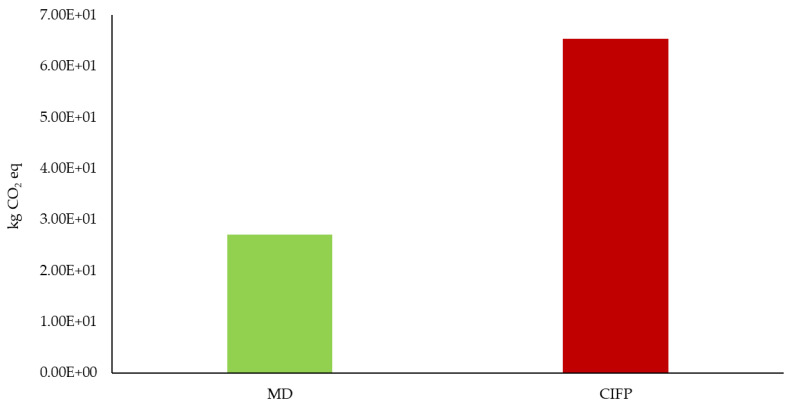
Carbon footprint of Mediterranean diet and Italian dietary habits (MD: Mediterranean diet; CIFP: Current Italian food patterns).

On the other hand, regarding CF, the results of which are expressed in Figure 5, it emerges that Italian eating habits generate a total CF of 6.54 × 10^1^ kg CO_2_ eq, +142% compared to the Mediterranean diet (2.7 × 10^1^ kg CO_2_ eq).

**Figure 6:**
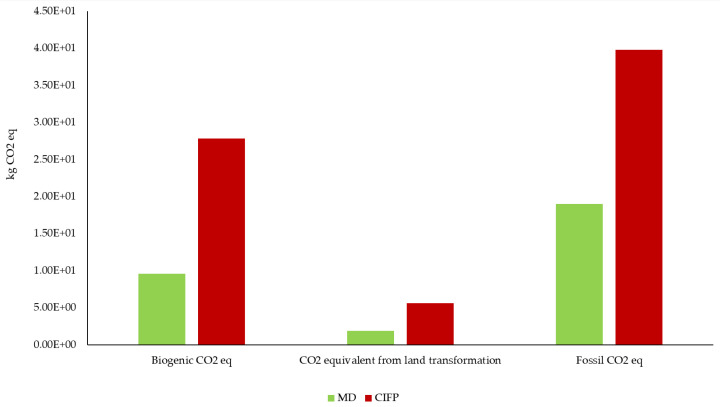
Greenhouse gas protocol results (MD: Mediterranean diet; CIFP: Current Italian food patterns).

Thus, in one year, following the Mediterranean diet could lead to a saving of 2.00 × 10^3^ kg CO_2_ eq per person. The higher CF of the CIFP can be attributable to climate-changing emissions due to increased fuel use of fishing vessels and maritime transport of goods, as well as deforestation and practices related to intensive livestock farming. As a result, Italian eating habits have a higher CF than the Mediterranean diet, making them less sustainable, confirming the findings of ReCiPe 2016 and CED. To investigate CF further and understand specifically from which processes CO_2_ eq is produced, data were analyzed using the greenhouse gas protocol, which divides carbon dioxide emissions into three categories: Biogenic CO_2_ eq (carbon emissions from biogenic sources such as plants and trees), CO_2_ eq from land transformation (transformation of forests into cropland), and fossil CO_2_ eq (carbon emissions from fossil fuels) (Figure 6).

The histogram shows that biogenic CO_2_ eq emissions are +191% for CIFP compared with MD, with 2.78 × 10^1^ kg CO_2_ eq for the former and 9.55 × 10^0^ kg CO_2_ eq for the latter. In particular, enteric emissions from ruminants and those from grain processing to obtain feed are the processes that contribute the most to GHG release in both models. This highlights the greater environmental impact of overconsumption of meat and animal products in the CIFP than in the MD guidelines. CO_2_ eq emissions from land transformation are 1.89 × 10^0^ kg for MD and 5.60 × 10 kg for CIFP (+196%). In both models analyzed, these emissions result from the deforestation of large areas of tropical crops. However, the greater impact of CIFP than MD is probably due to the production of fodder to meet the excessive consumption of meat and meat products. Finally, in terms of fossil CO_2_ eq emissions, the percentage difference between MD (1.90 × 10^1^ kg CO_2_ eq) and CIFP (3.97 × 10^1^ kg CO_2_ eq) is +109%, probably due to higher fuel emissions for food transportation, conversion of grains into fodder, and the use of fossil fuels to convert forests into arable land.

## 4. Discussion

Among the two food patterns considered, the processes causing the most significant impact are essentially related to livestock activities due to the excessive consumption of meat, dairy products (milk, yogurt, cheese), and animal products typical of Italian eating habits. For example, in terms of GWTE and GWFE, the activities that weigh the most are most likely to be methane (CH_4_) emissions from intensive livestock farming and carbon dioxide (CO_2_) emissions from the processing of grain for animal feed, as well as from the engines of ships that transport raw materials around the world, mainly from Brazil, India, the United States, and Argentina to Europe. Effects related to increased GW could be deaths, injuries, and post-traumatic stress events caused by improved frequency of adverse climate events or worsened living conditions due to disastrous climate events [36]. For ME, LU, and WCAE, however, the activities that weigh most heavily on these impact categories are the cultivation of cereals such as wheat and rice, which are also used to produce feed, while the intensive use of nitrates released by chemical fertilizers contributes to marine eutrophication (ME). At the same time, primary grain production for livestock leads to increased consumption of water resources (WCAE) for field irrigation and increased land use (LU) which is used almost exclusively for animal feed. However, increased consumption of meat and livestock products also affect increased nitrogen oxide (N_2_O) emissions from cereal-use fertilizers, fertilizers that end up in the atmosphere, contributing to stratospheric ozone depletion, as reflected, for example, in increased SOD, the health consequences of which are directly linked to increased UV-B radiation at the earth’s surface [37,38]. This also affects the release of NO_X_ into the atmosphere, as found in the category “Ozone formation, human health (OFHH)” (1.79 × 10^−7^ DALYs for CIFP and 1.07 × 10^−7^ DALYs for MD) as well as fine particulate matter, as evidenced by FPMF (+95% for CIFP), due to the release of NO_X_ into the air and PM_2_._5_ released mainly from diesel combustion of cargo ships and ammonium hydroxide (NH_4_OH) from pig farms. There are also other adverse effects related, for example, to the release of heavy metals in agricultural soils (HCT), particularly cadmium, caused by commercial sludge used in agriculture as field fertilizer and highly toxic to humans, or related to the elevated formation of O_3_, a potent oxidizing molecule that causes inflammation of the airways and lung tissues, increasing the incidence of lung cancer and asthma [39,40]. Thus, from the results of the LCA, CED, and CF, it emerges that overall Italian food consumption and habits are less sustainable than the LARN guidelines, mainly due to the high consumption of meat, fat, refined sugars, and convenience foods, and thus the transition of Italian food consumption patterns toward energy-intensive foods of animal origin causes significant environmental damage compared to a healthier and more balanced diet such as the MD. Therefore, following a Mediterranean diet is confirmed to be a more sustainable choice. Changing dietary patterns is necessary to improve the sustainability of diets to promote the environmental compatibility of food production and consumption, also in accordance with the European Farm to Fork strategy.

The results of our study are also confirmed by other studies in the literature, where different dietary patterns of consumption in different countries, including Germany [41], Portugal [13], the USA [42], and Spain [27,43,44], are compared with the Mediterranean diet. For example, Paris et al. (2022) [41] evaluated the sustainability of the German diet using an LCA approach, showing that most of the impacts are determined by the intake of animal products (including, for example, sausages and meat) and ready-to-eat, sugary drinks and alcohol, with the Mediterranean diet being more sustainable. Belgacem et al.’s (2021) [45] results show that a shift toward a Mediterranean-type dietary pattern can induce a saving of 10 m^2^/capita/day of land, 240 l/capita/day of water, and 3 kg of CO_2_/capita/day of GHG emission reduction compared to a European-type diet and a saving of 18 m^2^/capita/day of land, 100 l/capita/day of water, and 4 kg of CO_2_/capita/day of GHG emission reduction compared to a Western-type diet. Esteve-Llorens et al. (2020) [13] evaluated the carbon footprint of the Portuguese dietary pattern by proposing an example of a more sustainable alternative diet, with their results showing that the Portuguese dietary pattern results in an average CF of 4.20 kg CO_2_ eq/inhabitant/day, while the MD-like alternative diet leads to an increase in the nutritional quality of about +67% and a reduction in CF of about −25%. In Spain, three studies evaluate the environmental performance of different dietary models. Specifically, Martinez and Delgado (2020) [43] quantify the CF of some school menus following Spanish dietary guidelines (610 kcal/lunch-meal) by proposing a meatless menu that has a lower CF than a traditional menu. González-García et al. (2020) [44] compared a widespread and traditional Mediterranean diet (MD), the Southern European Atlantic diet (SEAD), and the dietary pattern recommended by the Spanish guidelines (NAOS), showing how GHGs emissions are lower for MD (2.79 kg CO_2_) than for NAOS (3.15) and SEAD (3.62), with MD generally performing the best. Similar results were also found by Batlle-Bayer et al. (2019) [27], in whose study the diet-related GHG emissions of current dietary patterns would be reduced by −17% and −11% when switching to NAOS and MD diets. Finally, Chapa et al. (2020) [42] evaluated the environmental performance of different dietary patterns in the U.S., comparing a typical one with those recommended by the U.S. Dietary Guidelines, including MD. Animal products, including meat and dairy, and discretionary foods were identified as the food categories with the greatest global warming potential, with MD being the most sustainable of the recommended diets. In general, however, it is worth noting that the environmental impacts and amounts of GHGs associated with a dietary food pattern varied between the individual choice of food products, and it depends mainly on the efficiency of the Food Supply Chain. Therefore, our study, as well as numerous studies available in the literature, reports how there are considerable differences between actual food consumption patterns and dietary recommendations. While the MD recommends a high consumption of fresh produce including fruits and vegetables, extra virgin olive oil, and very moderate consumption of red meat, data on actual food consumption patterns indicate a different reality associated with an increased intake of processed foods and resource-intensive products, such as those of animal origin, which implies resource intensification in the production chain and increased environmental impacts. The Mediterranean diet, therefore, in addition to being correlated with a low incidence of non-communicable diseases, mainly due to its high intake of vegetables, fruits, and cereals, low intake of animal fats, and moderate consumption of olive oil as the main source of fats, also implies lower environmental impacts than the Western dietary patterns prevalent in various countries, including Italy, as shown by the results of our study. Based on these results, it emerges how the transition to more balanced and relatively more sustainable food patterns such as MD, compared to current consumption patterns, in addition to improved population health, could help to pursue some ambitious goals, such as the SDGs (particularly Goals 2, 12, and 13), decarbonization, the European Green Deal (leading to the preservation of biodiversity and production from field to fork), and the European Farm to Fork strategy. For these reasons, it will be important for MD to inspire food policies from here on out, focusing consumption patterns on territoriality, seasonality, and zero kilometers. Thus, the main desirable changes for a healthy and sustainable diet should include a reduction in consumption of red and processed meat (−67/−90%), sugar (−85%), dairy products (−5%), and eggs (−40%). Protein intake should be promoted by the consumption of nuts and legumes, considering the beneficial effects of these foods on health. In addition, promoting nutrition education programs to improve the availability and accessibility of products with a better impact on human health and the environment could be the key to sustainable development. However, our analysis has several limitations that pose additional challenges and methodological limitations. First, taking as a functional unit the 14,000 kcal that should be consumed in a week (according to LARN recommendations), without considering the nutritional quality of the diets and thus without making assessments and distinctions on the nutrients (protein, fiber, vitamins A, C, E, minerals), provided by the food groups. Furthermore, some of the limitations of the LCIA values could be related to the lack of detailed information on harvesting dates for different crop products, as well as livestock farming. This limited information could represent a source of uncertainty that will require additional assessment. Another limitation may be represented by the non-consideration of early-age children, for comparison with middle and late age (18–74 years), to determine the timeframe to shift towards MD food habits. Therefore, to conduct a more in-depth analysis, it is necessary to also consider nutritional aspects, especially concerning current consumption, which is characterized by unbalanced meals in terms of both quantity and nutrients, which can lead to metabolic wastage of food, resulting in the accumulation of body fat that manifests itself in the form of obesity and/or overweight. Second, another limitation is that only long-term impacts on human health have been considered, without considering social consequences. Therefore, building on these limitations, it will be relevant to consider social impacts in an overall assessment of sustainability and nutritional quality of eating habits to better address the public health problem. These aspects will be analyzed in a future study.

## 5. Conclusions

The shift toward healthier and more sustainable diets is one of the most important challenges, both for the pursuit of national and international decarbonization goals and for the general improvement of population health. In this study, a comparative LCA was conducted between the environmental impacts related to the foods routinely consumed by Italians in 2019 and those that should be consumed according to MD guidelines. The results showed that the Italian dietary pattern, with high consumption of animal products and refined sugars, has a greater environmental impact than the Mediterranean dietary pattern. Specifically, the CIFP had a greater impact on the macro-areas Environment (+133%), Human Health (+100%), and Economics (+59%) than the MD model (with the latter likely to induce annual fossil and mineral resource savings of $53.35 per person, representing a total savings of $3.2 billion per year). The CED found that the MD is less energy-intensive than the CIFP for non-renewable energy commodity use categories, namely fossil (1.90 × 10^2^ MJ eq vs. 3.24 × 10^2^ MJ eq), nuclear (9.79 × 10^1^ MJ eq vs. 1, 78 × 10^2^ MJ eq), and biomass (9.90 × 10 MJ eq vs. 2.92 × 10^1^ MJ eq), and a shift toward MD would also lead to a substantial reduction in greenhouse gas emissions (annual savings of 2.00 × 10^3^ kg CO_2_ eq per person). This was due to the increased consumption of meat and meat products, both in terms of quantity and frequency, which require significant use of resources such as soil and water and result in greater emissions of pollutants into the biosphere. Specifically, processed meats are consumed 11 times more than recommended intake levels, red meats three times more, and white meats two times more. Milk and dairy products (yogurt, cheese, etc.) are consumed three times more than the guidelines, inevitably leading to more serious environmental and public health consequences. In conclusion, the results of our study show that Italian eating habits are less sustainable than the nutritional pattern recommended by the Mediterranean diet, and therefore a shift toward a Mediterranean dietary pattern could exert less pressure on the environment and human health.

## Figures and Tables

**Table 1:** Life cycle inventory of Mediterranean diet (MD) and current Italian food patterns (CIFP) in 2019.

INPUTS	UNIT	MD	CIFP
**Meat, fish, and eggs**			
Red meat	g	100	305
White meat	g	200	355
Processed meat	g	50	509
Fish and fish products	g	350	500
Eggs	g	150	238
**Milk and Dairy products**			
Milk and yogurt	g	1125	1037
Cheeses	g	250	390
**Cereal products**			
Bread	g	1225	728
Pasta	g	840	410
Sweet pastries (biscuits, cakes, croissants, etc.)	g	80	277
**Legumes**			
Dried legumes	g	150	157
**Fats and Oils**			
Extra virgin olive oil	g	210	135
**Fruits and vegetables**			
Fresh fruit	g	3150	1872
Vegetables	g	2800	2118
**Random foods**			
Sugar, sweets, snacks, alcohol-free beverages	g	70	479
Alcoholic beverages	g	686	676
Nuts	g	60	44
**OUTPUTS**	**UNIT**	**MD**	**CIFP**
Total Weekly Kcalories	Kcal	14,000	14,000

**Table 2:** Life cycle impact assessment results of ReCiPe 2016 Endpoint method.

Impact Categories	MD	CIFP
**Environmental (species/yr)**		
Global warming. Terrestrial ecosystem	8.28 × 10^−8^	1.88 × 10^−7^
Global warming. Freshwater ecosystem	2.26 × 10^−12^	5.12 × 10^−12^
Ozone formation. Terrestrial ecosystem	1.55 × 10^−8^	2.59 × 10^−8^
Terrestrial acidification	5.80 × 10^−8^	1.26 × 10^−7^
Freshwater eutrophication	3.28 × 10^−9^	6.47 × 10^−9^
Marine eutrophication	4.85 × 10^−11^	1.15 × 10^−10^
Terrestrial ecotoxicity	6.96 × 10^−10^	1.06 × 10^−9^
Freshwater ecotoxicity	4.67 × 10^−10^	6.65 × 10^−10^
Marine ecotoxicity	7.74 × 10^−11^	1.20 × 10^−10^
Land use	2.79 × 10^−7^	6.82 × 10^−7^
Water consumption. Terrestrial ecosystem	1.21 × 10^−8^	2.26 × 10^−8^
Water consumption. Aquatic ecosystem	1.99 × 10^−12^	4.43 × 10^−12^
TOTAL	4.52 × 10^−7^	1.05 × 10^−6^
**Human health (DALYs)**		
Global warming. Human health	2.75 × 10^−5^	6.22 × 10^−5^
Stratospheric ozone depletion	8.16 × 10^−8^	1.98 × 10^−7^
Ionizing radiation	4.30 × 10^−8^	7.27 × 10^−8^
Ozone formation. Human health	1.07 × 10^−7^	1.79 × 10^−7^
Fine particulate matter formation	3.86 × 10^−5^	7.53 × 10^−5^
Human carcinogenic toxicity	1.66 × 10^−6^	2.86 × 10^−6^
Human non-carcinogenic toxicity	7.22 × 10^−6^	1.07 × 10^−5^
Water consumption. Human health	1.82 × 10^−6^	2.31 × 10^−6^
TOTAL	7.70 × 10^−5^	1.54 × 10^−4^
**Economic (USD)**		
Mineral resource scarcity	3.01 × 10^−2^	6.61 × 10^−2^
Fossil resource scarcity	1.72 × 10^0^	2.71 × 10^0^
TOTAL	1.72 × 10^0^	1.72 × 10^0^

MD: Mediterranean diet; CIFP: Current Italian food patterns; DALYs: Disability-adjusted-life years; USD: US dollars.

**Table 3:** Economic impacts of the two dietary patterns (The impacts were calculated considering the functional unit of 14.000 kcal/weekly, which is the LARN-recommended weekly caloric intake for a normal-weight adult).

Impact Categories	MD	CIFP	Δ = CIFP − MD
Mineral resource scarcity	0.03 $	0.07 $	0.04 $
Fossil resource scarcity	1.72 $	2.71 $	0.99 $
Total	1.75 $	2.78 $	1.03 $
Annual costs	91.01$	144.36$	53.35$

MD: Mediterranean diet; CIFP: Current Italian food patterns.

**Table 4:** Results (MJ eq) of cumulative energy demand.

Impact Categories	Mediterranean Diet	Current Consumption
Non-renewable: fossil	1.90 × 10^2^ MJ eq	3.24 × 10^2^ MJ eq
Non-renewable: nuclear	9.79 × 10^1^ MJ eq	1.78 × 10^2^ MJ eq
Non-renewable: biomass	9.90 × 10^0^ MJ eq	2.92 × 10^1^ MJ eq
Renewable: biomass	3.22 × 10^2^ MJ eq	9.13 × 10^2^ MJ eq
Renewable: water	8.59 × 10^0^ MJ eq	1.56 × 10^1^ MJ eq
Renewable: wind, solar, geothermal	2.36 × 10^0^ MJ eq	3.80 × 10^0^ MJ eq

## Data Availability

All data are publicly available and cited within the text in accordance with journal guidelines.

## References

[1] Shukla P.R., Skeg J., Buendia E.C., Masson-Delmotte V., Pörtner H.O., Roberts D.C., Zhai P., Slade R., Connors S., Van Diemen S., IPCC (2019). Climate Change and Land: An IPCC Special Report on Climate Change, Desertification, Land Degradation, Sustainable Land Management, Food Security, and Greenhouse Gas Fluxes in Terrestrial Ecosystems.

[2] Galli A., Iha K., Halle M., El Bilali H., Grunewald N., Eaton D., Capone R., Debs P., Bottalico F. (2017). Mediterranean countries’ food consumption and sourcing patterns: An Ecological Footprint viewpoint. Sci. Total Environ..

[3] Serafini M., Toti E. (2016). Unsustainability of Obesity: Metabolic Food Waste. Front. Nutr..

[4] FAOSTAT (2019). Food and Agriculture Data. http://www.fao.org/faostat/en/#data.

[5] NCD Risk Factor Collaboration (NCD-RisC) (2021). Heterogeneous contributions of change in the population distribution of body mass index to change in obesity and underweight. eLife.

[6] Danaei G., Singh G.M., Paciorek C.J., Lin J.K., Cowan M.J., Finucane M.M., Farzadfar F., Stevens G.A., Riley L.M., Lu Y. (2013). The global cardiovascular risk transition: Associations of four metabolic risk factors with national income, urbanization, and western diet in 1980 and 2008. Circulation.

[7] Castaldi S., Dembska K., Antonelli M., Petersson T., Piccolo M.G., Valentini R. (2022). The positive climate impact of the Mediterranean diet and current divergence of Mediterranean countries towards less climate-sustainable food consumption patterns. Sci. Rep..

[8] Gephart J.A., Davis K.F., Emery K.A., Leach A.M., Galloway J.N., Pace M. (2016). The environmental cost of subsistence: Optimizing diets to minimize footprints. Sci. Total Environ..

[9] Vitale M., Giosuè A., Vaccaro O., Riccardi G. (2021). Recent trends in dietary habits of the Italian population: Potential impact on health and the environment. Nutrients.

[10] Willett W., Rockstrom J., Loken B., Springmann M., Lang T., Vermeulen S., Garnet T., Tilman D., De Clerk F., legno A. (2019). Food in the Anthropocene: The EAT-Lancet Commission on healthy diets from sustainable food systems. Lancet.

[11] Castañé S., Antón A. (2017). Assessment of the nutritional quality and environmental impact of two food diets: A Mediterranean and a vegan diet. J. Clean. Prod..

[12] Walker C., Gibney E.R., Hellweg S. (2018). Comparison of Environmental Impact and Nutritional Quality among a European Sample Population—Findings from the Food4Me study. Sci. Rep..

[13] Esteve-Llorens X., Dias A.C., Moreira M.T., Feijoo G., Gonzalez-Garcia S. (2020). Evaluating the Portuguese diet in the pursuit of a lower carbon and healthier consumption pattern. Clim. Chang..

[14] González-García S., Esteve-Llorens X., Moreira M.T., Feijoo G. (2018). Carbon footprint and nutritional quality of different human dietary choices. Sci. Total Environ..

[15] Keys A., Menotti A., Karvonen M.J., Aravanis C., Blackburn H., Buzina R., Djordjevic B.S., Dontas A.S., Fidanza F., Keys M.H. (1986). The diet and 15-year death rate in the seven countries study. Am. J. Epidemiol..

[16] Naja F., Itani L., Hamade R., Chamieh M.C., Hwalla N. (2019). Mediterranean diet and its environmental footprints amid nutrition transition: The case of Lebanon. Sustainability.

[17] SINU (2018). LARN—Livelli di Assunzione di Riferimento di Nutrienti ed Energia per la Popolazione Italiana—IV Revisione. https://sinu.it/larn/.

[18] European Commission Farm to Fork Strategy. For a Fair, Healthy and Environmentally Friendly Food System. https://food.ec.europa.eu/horizontal-topics/farm-fork-strategy_en.

[19] European Commission (2021). European Green Deal. https://ec.europa.eu/info/strategy/priorities-2019-2024/european-green-deal/delivering-european-green-deal_en.

[20] United Nations. Department of Social Affairs (2015). Transforming Our World: The 2030 Agenda for Sustainable Development. https://sdgs.un.org/goals.

[21] Coldiretti (2020). Consumi in Italia. https://www.coldiretti.it/.

[22] Ismea (2020). Consumi Alimentari. I Consumi Domestici delle Famiglie Italiane. http://www.ismeamercati.it/flex/cm/pages/ServeBLOB.php/L/IT/IDPagina/3562.

[23] Istituto Nazionale di Statistica (2020). Spese per Consumi. Spesa Media Mensile Familiare (in Euro Correnti). http://dati.istat.it/viewhtml.aspx?il=blank&vh=0000&vf=0&vcq=1100&graph=0&view-metadata=1&lang=it&QueryId=17912.

[24] Ruggieri R., Vinci G., Ruggeri M., Sardaryan H. (2020). Food losses and food waste: The Industry 4.0 opportunity for the sustainability challenge. Rev. Stud. Sustain..

[25] (2006). Principles and framework of Life Cycle Assessment.

[26] (2006). Environmental Management—Life Cycle Assessment—Requirements and Guidelines.

[27] Batlle-Bayer L., Bala A., García-Herrero I., Lemaire E., Song G., Aldaco R., Fullana-i-Palmer P. (2019). The Spanish Dietary Guidelines: A potential tool to reduce greenhouse gas emissions of current dietary patterns. J. Clean. Prod..

[28] Veeramani A., Dias G.M., Kirkpatrick S.I. (2017). Carbon footprint of dietary patterns in Ontario, Canada: A case study based on actual food consumption. J. Clean. Prod..

[29] van de Kamp M.E., van Dooren C., Hollander A., Geurts M., Brink E.J., van Rossum C., Biesbroek S., de Valk E., Toxopeus I.B., Temme EH M. (2018). Healthy diets with reduced environmental impact?—The greenhouse gas emissions of various diets adhering to the Dutch food-based dietary guidelines. Food Res. Int..

[30] Colomb V., Ait Amar S., Mens C.B., Gac A., Gaillard G., Koch P., Mousset J., Salou T., Tailleur A., van der Werf H.M.G. (2015). AGRIBALYSE^®^, the French LCI database for agricultural products: High-quality data for producers and environmental labeling. OCL.

[31] Wernet G., Bauer C., Steubing B., Reinhard J., Moreno-Ruiz E., Weidema B. (2016). The ecoinvent database version 3 (part I): Overview and methodology. Int. J. Life Cycle Assess..

[32] Nemecek T., Bengoa X., Lansche J., Roesch A., Faist-Emmenegger M., Rossi V., Humbert S. (2019). Methodological Guidelines for the Life Cycle Inventory of Agricultural Products. Version 3.5, December 2019. World Food LCA Database (WFLDB).

[33] Huijbregts M. (2016). ReCiPe 2016—A Harmonized Life Cycle Impact Assessment Method at Midpoint and Endpoint level. Report I: Characterization. National Institute for Public Health and the Environment. https://www.rivm.nl/bibliotheek/rapporten/2016-0104.pdf.

[34] Afshin A. (2019). Health effects of dietary risks in 195 countries, 1990–2017: A systematic analysis for the Global Burden of Disease Study 2017. Lancet.

[35] Forster P., Artaxo P., Solomon S., Qin D., Manning M., Chen Z., Marquis M., Averyt K.B., Tignor M., Miller H.L. (2007). Changes in Atmospheric Constituents and in Radiative Forcing. Climate Change 2007: The Physical Science Basis.

[36] Trisos AC H., Merow C., Pigot A.L. (2020). The projected timing and abrupt ecological disruption from climate change. Nature.

[37] Häder D.P., Williamson C.E., Wangberg S.A., Rautio M., Rose K.C., Gao K., Helbling E.W., Sinha R., Worrest R. (2015). Effects of UV radiation on aquatic ecosystems and interactions with other environmental factors. Photochem. Photobiol. Sci..

[38] Dowlat MJ H., Karuppannan S.K., Sinha P., Dowlath N.S., Arunachalam K.D., Ravindran B., Chang S.W., Nguyen-Tri P., Nguyen D.D. (2021). Effects of radiation and role of plants in radioprotection: A critical review. Sci. Total Environ..

[39] Lippmann M. (1989). Health Effects of Ozone. A Critical Review. J. Air Pollut. Control Assoc..

[40] Hatch G.E., Duncan K.E., Diaz-Sanchez D., Schmitt M.T., Ghio A.J., Carraway M.S., McKee J., Dailey L.A., Berntsen J., Devlin R.B. (2014). Progress in assessing air pollutant risks from In vitro exposures: Matching ozone dose and effect in human airway cells. Toxicol. Sci..

[41] Paris JM G., Falkenberg T., Nöthlings U., Heinzel C., Borgemeister C., Escobar N. (2022). Changing dietary patterns is necessary to improve the sustainability of Western diets from a One Health perspective. Sci. Total Environ..

[42] Chapa J., Farkas B., Bailey R.L., Huang J. (2020). Evaluation of the environmental performance of dietary patterns in the United States considering food nutrition and satiety. Sci. Total Environ..

[43] Martinez S., del Mar Delgado M., Martinez Marin R., Alvarez S. (2020). Carbon footprint of school lunch menus adhering to the Spanish dietary guidelines. Carbon Manag..

[44] González-García S., Green R.F., Scheelbeek P.F., Harris F., Dangour A.D. (2020). Dietary recommendations in Spain—Affordability and environmental sustainability?. J. Clean. Prod..

[45] Belgacem W., Mattas K., Arampatzis G., Baourakis G. (2021). Changing Dietary Behavior for Better Biodiversity Preservation: A Preliminary Study. Nutrients.

